# The Association of Longer Breastfeeding Duration and Socioeconomic, Pregnancy, Childbirth and Postpartum Characteristics

**DOI:** 10.3390/medicina60050792

**Published:** 2024-05-10

**Authors:** Jekaterina Kozachenko, Anda Kivite-Urtane, Frederika Berzina, Ieva Evelina Stolcere, Gunta Lazdane

**Affiliations:** 1 Institute of Public Health, Riga Stradins University, Kronvalda Boulevard 9, LV-1010 Riga, Latvia; 2 Department of Public Health and Epidemiology, Riga Stradins University, Kronvalda Boulevard 9, LV-1010 Riga, Latvia; 3 Department of Obstetrics and Gynaecology, Riga Stradins University, Miera iela 45, LV-1013 Riga, Latvia

**Keywords:** breastfeeding duration, associated factors, smoking, prenatal classes, early breastfeeding initiation

## Abstract

*Background and Objectives:* Breastmilk is the safest and most suitable food for an infant, playing the role of their first vaccine and containing all the essential nutrients for the first months of life. The World Health Organisation recommends exclusive breastfeeding for the first 6 months of life and continued breastfeeding while introducing a child to complementary foods until 2 years and beyond. According to Latvian statistics from 2022, only 27.4% of babies were breastfed for 12 months. The aim of this study was to determine the socio-economic factors and factors related to pregnancy, childbirth and postpartum that influence breastfeeding for more than 6 months in Latvia. *Materials and Methods*: Data were used from the cross-sectional survey “Research on factors and behaviours affecting the sexual and reproductive health of the population of Latvia”, which was conducted in 2023. A study sample was randomised and stratified by gender and five age groups. The analyses in this study are based on a sample of women who had given birth at least once (*n* = 1407), and the dependent variable was the duration of breastfeeding their last child. Binary logistic regression was conducted to identify the associated factors. *Results:* The point prevalence of longer duration of breastfeeding for the last child was 47.9% (*n* = 674). The odds of longer breastfeeding duration were higher among mothers who did not smoke during pregnancy (vs. smokers, aOR 2.1, *p* < 0.001), of Latvian nationality (vs. Russian, aOR 1.3, *p* = 0.03), who had two childbirth (vs. one, aOR 1.5, *p* = 0.003), who had the highest level of education (vs. primary education, aOR 2.0, *p* = 0.03), started breastfeeding immediately after the birth (vs. later than the first day, aOR 1.7, *p* = 0.01) or on the first day (vs. later, aOR 1.6, *p* = 0.01). *Conclusions*: We documented socio-demographic pregnancy and childbirth factors associated with longer breastfeeding durations. Efforts to promote breastfeeding practices should target mothers from the most vulnerable groups.

## 1. Introduction

Breastmilk is the safest and most suitable food for an infant, playing a role as their first vaccine and containing all the essential nutrients, antibodies, hormones and antioxidants for the first months of life [1,2]. Furthermore, it continues to provide up to half or more of a child’s nutritional needs during the second half of the first year, and up to one-third during the second year of life [3]. Breastfed babies are less likely to suffer from dental malocclusion and infections such as gastrointestinal, urinary tract, pneumonia and acute otitis media [4]. Breastfeeding is also associated with increased intelligence and, possibly, reductions in overweight and diabetes [5,6]. Likewise, a woman who breastfeeds has several health benefits, including short-term benefits (such as reduced risk of postpartum haemorrhage) and long-term benefits (such as reduced risk of breast and ovarian cancer) [6,7].

The World Health Organisation (WHO) recommends that exclusive breastfeeding should be ensured from birth until 6 months of age, while continued breastfeeding while introducing a child to complementary feeding at 6 months until 2 years and above is also recommended [8]. Similar recommendations are given by the Latvian Ministry of Health, which emphasises that, in addition to complementary feeding, it is essential to continue breastfeeding until at least 1 year of age. If the mother is willing, breastfeeding can continue until the baby is 2 years old or more [9]. The WHO European Region has the lowest rates of exclusive breastfeeding at 6 months of age in the world, with a prevalence of about 25% [10]. According to the Health Statistics of Latvia in 2022, 88.1% of infants were breastfed for 6 weeks, 53.7% for 6 months, 27.4% were breastfed for 12 months, and only 16.1% of children under 6 months were exclusively breastfed [11]. In 2012, the World Health Assembly (WHA) approved a Comprehensive Implementation Plan for Maternal, Infant, and Young Child Nutrition, which set six global targets to be achieved by 2025, including increasing the rate of exclusive breastfeeding for the first 6 months to at least 50%.

Many factors influence the duration of breastfeeding, including planned pregnancy, lifestyle factors of the mother, antenatal preparation (informing pregnant women about the benefits and management of breastfeeding), early initiation of breastfeeding within the first hour after birth, as well as positive social norms that support and encourage breastfeeding (including at work, in public spaces) that serve to empower mothers to breastfeed, etc. [12,13,14]. In terms of the longer duration of breastfeeding in Latvia and other Baltic countries, there is a lack of information regarding the impact of mentioned factors. Furthermore, a majority of studies usually are focused on exclusive breastfeeding duration, while this study focuses on a longer duration, as receiving any amount of breastmilk is essential for the baby. In addition, the study data were gathered from only the fourth nationwide research on factors and behaviours affecting the sexual and reproductive health of the population of Latvia since 1998, which is very important for both researchers from the Baltic region and policy makers. This study shows different correlations regarding the association of the presence of the partner at birth, their psychological support after childbirth and the role of prenatal classes and breastfeeding, than in another studies. That makes novelty of these study and could be explained, that the role of the partner and prenatal classes are not sufficient for the Latvian women during the breastfeeding period.

Breastfeeding is also a big part of the 2030 Agenda for Sustainable Development, as it is linked to several of the Sustainable Development Goals (SDGs) due to its impact on various aspects of individual and societal wellbeing. Breastfeeding should be a priority when national governments around the world develop budgets and action plans to achieve SDGs [15].

According to the World Bank data of 2023, Latvia is a high-economy country [16] and has featured breastfeeding promotion programs during the last 20 years. However, according to statistical data, the breastfeeding durations in Latvia and Europe do not meet the recommendations of the WHO. This study provides information about the associated factors of longer breastfeeding duration that could help to develop more targeted breastfeeding promotions and improvements for policy makers not only in Latvia but also in other high-economy countries.

The aim of this study was to investigate the duration of breastfeeding in Latvia in relation to maternal socio-demographic, pregnancy, delivery and postpartum factors.

## 2. Materials and Methods

### 2.1. Study Design

We used the data from the quantitative cross-sectional study “Research on factors and behaviour affecting the sexual and reproductive health of the population of Latvia”, which was implemented within the framework of the procurement of the Ministry of Health of the Republic of Latvia (No. VM 2019/18/ESF). This is the fourth study conducted in Latvia on the sexual and reproductive health of the population (the previous studies were conducted in 1997, 2003 and 2011).

### 2.2. Ethical Consideration

The study protocol was approved by the Ethics Committee of Riga Stradins University, which conducted the study (protocol number 6-1/06/11, 28 May 2020). The first page of the questionnaire contained information about the research objectives and also informed the respondent that participation in the research was voluntary and that the information obtained would be anonymous. For adult responders (of age 18 years or more), filling out the questionnaire was considered to be consent to participating in the study. A separate written informed consent form was signed by the parents or caregivers in cases of underage responders.

### 2.3. Sampling Procedure

The sample of the study was randomised and stratified by gender and five age groups (15–19, 20–29, 30–39, 40–49, and 50–64 years), and the distribution of the population by nationality and urbanisation were considered to ensure nationally representative data. A sampling of 4181 respondents was achieved (2014 men and 2167 women), which is 0.2% of the 1.24 million residents of Latvia (aged 15–64 years), which formed the target population in 2018.

A stratified sample of households was randomly selected by computer generation from the address register administered by the State Land Service of Latvia. The households to be interviewed were selected using the algorithm of the so-called random route method, i.e., starting with the initial (starting) address, every second household in urban and semi-urban areas and every household in rural areas were approached. In every household, only one person was interviewed according to the principle of “Younger Man.” If the initial contact was not made (the residents of the household were not available), the household was visited up to three times before being marked as not reachable. A similar random-route method has been used more than 12 times in large population-based studies in Latvia before.

The analyses in this study are based only on a sample of women aged 15–64 who had given birth at least once (*n* = 1407) between the ages of 15 and 64.

### 2.4. Data Collection

Data collection took place between September 2020 and May 2023. Twenty-five specially trained interviewers randomly recruited respondents at their place of residence and administered a self-filled questionnaire in Latvian or Russian according to the respondent’s choice. After completing the questionnaires, respondents placed them in a self-adhesive envelope in the presence of the interviewer. The envelopes were opened only for data entry. The questionnaires were anonymous. The response rate was estimated at 65.3% (4181 valid questionnaires from 6400 invited individuals).

The content of the research questionnaires was piloted in thirty interviews. The purpose of piloting the questionnaire was to measure the perception and comprehensibility of the questionnaire to make corrections to the questions or structure of the questionnaire if necessary.

### 2.5. Measures

The outcome (dependent variable) was the duration of breastfeeding for the last child. Mothers were asked: “How long did you breastfeed at all?”

The independent variables of the study were grouped according to the socio-demographic, pregnancy, delivery and postpartum factors of the most recent pregnancy:

- Socio-demographic factors included questions on the mother’s nationality (Latvian, Russian, other), monthly net income per household member in the last six months (0–500, 501–800, ≥801 euro), number of births (one, two, three and more) and mother’s level of education (primary—9 years of schooling, general or vocational secondary—12 or 13 years of schooling, university or college). The question “In what year was your last birth?” determined the variable ‘Period of last birth’. The responses were coded into two categories: “1970–1999” and “2000–2023”, because the first Latvian “Baby—friendly hospital initiative” was developed in 2000 by the first Breastfeeding promotion commission of the Ministry of Health of Latvia.
- Pregnancy factors included questions about smoking during pregnancy, with the options of answers being “yes” or “no”. The respondents were asked about the antenatal care and attending prenatal classes during pregnancy (with the options of answers being “yes” or “no”), having gestational diabetes mellitus and/or hypertension during pregnancy (yes/no), and planning of last pregnancy, i.e., “Did you plan your last pregnancy?” with the categorisation of responses being “yes” or “no”.
- Childbirth factors were described as the initiation of breastfeeding (immediately after birth/on the first day/later) and the presence of a partner during labour.
- Postpartum factors prompted the question “Did you need emotional or psychological support in the postpartum period?” Psychological support from a partner was determined by the questions “Did you receive your partner’s emotional or psychological support; did he try to understand and support you in postpartum period?”

### 2.6. Statistical Analysis

Data analysis was carried out using SPSS IBM Statistics v.27.0 (IBM corp., Armonk, NY, USA). The data were weighted according to the general population of Latvia on the basis of four characteristics: gender, five age groups (15–19, 20–29, 30–39, 40–49, 50–64 years), type of settlement (Riga, other city, rural area) and nationality (Latvian, non-Latvian) according to the data of the Office of Citizenship and Migration Affairs of the Republic of Latvia.

The information provided by the national register of newborns of Latvia showed, that the duration of breastfeeding decreased almost twice after a baby reached 6 months [11]. Therefore, the continued dependent variable was categorised as a dichotomous variable for understanding the reason and which factors are associated with that tendency.

Descriptive statistics (frequencies, cross tabulations, medians) were used to describe the study sample. The statistical significance of differences in the distribution of dependent variables between the strata of independent variables was detected using the chi-square test. Binary logistic regression was used to calculate the odds ratios (ORs) for both the univariate and multivariate analyses. The collinearity between factors was checked before developing a multivariate regression model. No collinearity was identified; thus variables with statistically significant associations in univariate analyses (*p* < 0.05) (nationality of mother, year of the last labour, number of the childbirths, maternal educational level, smoking during pregnancy, attendance of the prenatal classes, breastfeeding for the first time, partner’s presence on a labour) were included in a binary logistic regression model. All results are reported as ORs with 95% confidence intervals (CI). Statistical significance was considered as *p* < 0.05.

## 3. Results

The data from 1407 women aged 15–64 years who had experience of childbirth were analysed. The first group included mothers who breastfed their last baby between 0 and 6 months—52.1% (*n* = 733), while the second group involved mothers who breastfed their baby 7 and more months—47.9% (*n* = 674). The mean duration of breastfeeding was 8.7 (SD—7.8) (min.: 0–max.: 60) months, while the median duration was 6.0 (interquartile range: 3.0–12.0) months.

Table 1 describes the respondents’ socio-demographic, pregnancy, childbirth and postpartum characteristics related to breastfeeding duration. The first group of mothers (breastfeeding 0–6 months) had a median duration of breastfeeding—3 months, while the second group of mothers (7+ months) had a median breastfeeding period—12 months.

Although more than half of the women in both groups were Latvians, the first group of mothers (breastfeeding 0–6 months) had a significantly higher proportion of non-Latvians (34.1%, *n* = 250). Respondents with breastfeeding experience of 0–6 months had a statistically higher proportion of women who had their last birth in the period 1970–1999 (40.2%, *n* = 291). The group with shorter breastfeeding duration also had a higher proportion of women with lower income levels (0–500 euro, 63.9%, *n* = 418), with experience of one childbirth (36.0%, *n* = 264), had general primary, secondary/vocational secondary educations (55.5%, *n* = 406), who smoked during pregnancy (14.4%, *n* = 104), did not attend prenatal classes (76.5%, *n* = 534), started breastfeeding late (16.1%, *n* = 117) and experienced labour without the presence of a partner (78.3%, *n* = 573) in comparison to the second group.

There were no statistically significant differences between the two groups in terms of pregnancy planning, the incidence of gestational diabetes and/or hypertension during pregnancy, psychological support from a partner and the mothers’ need of psychological support during postpartum period.

The univariate binary logistic regression analyses (see Table 2) revealed that the odds of a longer breastfeeding period (7+ months) were significantly higher in the group of mothers of Latvian nationality (vs. Russian, OR 1.4 (95% CI 1.1–1.8), *p* = 0.004) and those who had their last labour in 2000–2023 (vs. 1970–1999, OR 1.6 (95% CI 1.3–1.9), *p* < 0.001). Higher odds of longer breastfeeding were detected in respondents with two childbirths vs. one (OR 1.5 (95% CI 1.2–1.9), *p* = 0.002). According to the crude analysis, having the highest educational level (university or college degree) was also associated with higher odds of a longer breastfeeding period vs. women with primary education (OR 3.2 (95% CI 1.9–5.4), *p* < 0.001), as was the group of non-smokers during pregnancy vs. smokers (OR 2.8 (95% CI 1.9–4.0), *p* < 0.001). Higher odds of longer breastfeeding were detected in respondents who attended prenatal classes during pregnancy vs. did not attend (OR 1.6 (95% CI 1.2–1.9), *p* < 0.001) as well as those whose partners have been with them during labour vs. did not (OR 1.8 (95% CI 1.4–2.3), *p* < 0.001). Additionally, according to the univariate analysis, the odds of longer breastfeeding were particularly high in the group who started breastfeeding immediately after giving birth (vs. later than on the first day, OR 2.0 (95% CI 1.5–2.9), *p* < 0.001) as well as when breastfeeding was started on the first day (vs. later than the first day, OR 1.6 (95% CI 1.1–2.3), *p* = 0.01). Interestingly, postpartum characteristics, such as psychological support from the partner after the birth and the mother’s need for psychological support, did not show statistically significant results.

After adjustment for all statistically significant independent variables, three socio-demographic characteristics remained statistically significantly associated with the longer breastfeeding period, i.e., the Latvian nationality of mothers (vs. Russian, aOR 1.3 (95% CI 1.0–1.7), *p* = 0.03) and other nationality (vs. Russian, aOR 1.5 (95% CI 1.0–2.2), *p* = 0.04), experience of two childbirths (vs. one, aOR 1.5 (95% CI 1.1–1.9), *p* = 0.003) and highest education group (vs. primary, aOR 2.0 (95% CI 1.0–3.7), *p* = 0.03). Non-smoking during pregnancy remained significantly associated with longer breastfeeding. (vs. smoking, aOR 2.1 (95% CI 1.4–3.3), *p* < 0.001). Breastfeeding initiation immediately after childbirth vs. later than on the first day (aOR 1.7 (95% CI 1.1–2.4), *p* = 0.01) and on the first day (vs. later (aOR 1.6 (95% CI 1.2–2.4), *p* = 0.01) had a significant association with longer breastfeeding.

## 4. Discussion

There is a lack of data about the influence of socio-economic factors and factors directly linked to pregnancy, delivery and the postpartum period on the duration of breastfeeding among women living in Latvia. Conducted in a large, representative population-based sample, our research provides information about the point prevalence of longer breastfeeding duration in association with different factors.

The results of our study on the duration of breastfeeding are quite similar to the results of the study conducted by the Latvian Ministry of Health in 2018, which showed that the average duration of breastfeeding was 8.3 months (in our case it is 8.7 months) [17]. It can be concluded that the duration of breastfeeding in Latvia does not meet the recommendations of both the WHO and the Ministry of Health of Latvia [2,8,18].

A number of studies have analysed the influence of socio-economic status (SES) on the duration of breastfeeding, focusing on maternal educational level, maternal unemployment benefit, social welfare and equivalent disposable income, confirming that low SESs are a risk factor for shorter breastfeeding even in countries with well-established social welfare support systems and positive breastfeeding traditions, such as Sweden [19,20,21,22]. In our study, we have also shown that mothers with higher SESs in Latvia breastfeed for longer. This result could be explained by the fact that maternity employment leads to the cessation of breastfeeding and that women with lower SESs have to start working earlier.

In addition, our study shows that mothers of Russian nationality are less likely to breastfeed for longer than mothers of the Latvian nationality. The Central Statistical Bureau of Latvia provides the information that 29.86% of the population of Latvia are Russian speaking people, while the share of people (aged 25–64) who do not know Latvian is 10.7% [23]. Due to the geopolitical situation, Latvian is the only language of communication with society, e.g., health promotion information and receiving health care. Therefore, information about health topics including prenatal classes often do not reach this part of the country’s population. As a result, the Latvian economy “will suffer” more due to people with low health literacy in the future.

The framework and recommendations for the organisation, funding and monitoring of future health promotion activities and programmes at national level, led by the Ministry of Health of Latvia, identify breastfeeding promotion for populations at risk of poverty and social exclusion as one of the key issues in the field of healthy nutrition in the community [24]. In addition, breastfeeding women in Latvia are supported by the government with free electronic information materials and information campaigns on breastfeeding [25]. In terms of multisectoral cooperation between the national and local levels, the majority of municipalities have provided free childbirth and breastfeeding education to young parents at least once a year over the past six years. Women with any SES can get free help from a volunteer breastfeeding counsellor in any region of Latvia [26]. Thus, in Latvia, various initiatives to promote breastfeeding are being introduced, but they do not seem to reach women with a lower socio-economic status, and new and specific approaches need to be sought to reach women at risk of social exclusion and Russian-speaking women.

Our study shows that mothers with two children breastfed their last child longer than mothers with one child. A 2018 study in Denmark also showed that primiparity is a factor associated with early breastfeeding problems (in parallel with lower self-efficacy and lower self-reported knowledge about breastfeeding, which may contribute to shorter breastfeeding duration in mothers with one baby) [27]. A 2015 study by Hackamn et al. shows that primiparous women had shorter intended breastfeeding durations, a greater delay from delivery to first breastfeeding attempt, were less likely to feed at least eight times in the first 24 h and had more breastfeeding problems during their maternity stay. These variables are likely to have contributed to the finding that primiparous women had more mixed formula and breastmilk feeding at hospital discharge, delayed lactogenesis, were less likely to achieve their intended breastfeeding goal and had shorter breastfeeding durations [28]. A study by Lindblad et al. in 2022 shows that primiparous women had a higher relative risk of having doubts about breastfeeding after discharge than multiparous women. At one and six weeks postpartum, twice as many primiparous women felt anxious or depressed as multiparous women [29]. There was more consistency for continued breastfeeding, with most studies showing a positive association between continued breastfeeding and multiparity [30].

These findings underline the importance of support and education for primiparous women by healthcare practitioners, organisations and members of their own families to promote breastfeeding continuation. Support options differ from one country to another. In Latvia, breastfeeding counselling is available not only from health professionals such as doctors, nurses and midwives, but also from other trained professionals like doulas, pep-moms and lactation consultants [31,32]. However, our study shows that this support for women who have given birth for the first time could be strengthened in our country.

Our study observes positive associations between non-smoking status during the pregnancy and breastfeeding duration. A 2018 study by Cohen et al. supports our findings, showing that, in addition to women who smoke throughout pregnancy, 50–80% of women who quit during pregnancy relapse to smoking within the first 6 months postpartum, and that smoking among breastfeeding women are associated with both shorter duration and lower milk production [30]. In addition, smoking during lactation is associated with higher rates of early cessation due to the effects of nicotine on milk volume [33]. Many smoking mothers believe that their milk contains nicotine from tobacco, which could be more harmful to their new-born than formula; therefore, smoking mothers have lower breastfeeding rates [34]. Additionally, some women think that formula is better and more convenient than breastfeeding due to widespread marketing [35]. Special support groups for pregnant women and young parents who smoke should be organised to help them quit smoking in Latvia.

According to WHO and UNICEF, one of the actions that support breastfeeding are infant feeding counselling, including antenatal and postnatal care [36]. A study conducted in 2024 by Oggero et al. shows that prenatal breastfeeding education can potentially influence breastfeeding duration when psychological components are integrated and supplemented with face-to-face postnatal support [37]. Our univariate analysis showed that attending antenatal classes was statistically significantly associated with longer breastfeeding duration; however, after adjustment the results remained statistically insignificant. Our results may be explained by the fact that, in Latvia, although the Cab. Reg. No. 611 “Procedure for Providing Maternity Assistance” states that medical staff should educate mothers about the importance of breastfeeding and help them with the correct technique, there is no regulation on how exactly this should be conducted [38]. There are different types of antenatal classes available, both free and paid for, but, again, there is no regulation on the uniform content of these classes. In our study, we did not ask women how many classes they had attended during pregnancy or whether they had attended antenatal classes where breastfeeding was discussed.

WHO and UNICEF recommend that infants are breastfed within the first hour, which is very important for the survival of newborns and the establishment of long-term breastfeeding [35]. Early initiation of breastfeeding protects newborns from infection and therefore reduces the risk of neonatal mortality from diarrhoea and other infections [39].

The Health Statistics Database of the Centre for Disease Prevention and Control in Latvia does not have records on the duration of breastfeeding initiation [11]. Our study provides important information on the prevalence of breastfeeding initiation in Latvia, reporting that longer breastfeeding duration is associated with early breastfeeding initiation. These findings are supported by WHO recommendations for breastfeeding [39].

In this study we wanted to analyse the duration of breastfeeding in relation to the year of the last birth. Historically, the Millennium Development Goals (MDGs) of 2000 set 15-year targets for significant improvements in wellbeing and development, with nutrition being the first objective [40]. In addition, Latvia’s first Baby-Friendly Hospital Initiative (BFHI) was developed in 2000 and involved 12 hospitals. The BFHI core purpose is to ensure that mothers and newborns receive timely and appropriate care, which includes establishment of optimal feeding of the new-born. The BFHI model is based on 10 steps to successful breastfeeding [41]. The results of the univariate analysis in this study show that the duration of breastfeeding was longer between 2000 and 2023 than between 1970 and 1999. Similar findings were described by Oliviera et al., who said that the promotion, protection and support of maternal breastfeeding through legislation, various standards and programmes adopted by the government were favourable to longer breastfeeding duration [42]. We recommend that all maternity units in Latvia promote and implement the essential strategic and practical changes needed to achieve Baby Friendly Hospital Initiative status, as it promotes and supports breastfeeding.

We suggest that proper management of breastfeeding education and support could increase the duration of breastfeeding in Latvia. In addition, antenatal classes should all follow the same guidelines and criteria. This will ensure high quality and reliability in the information provided in these classes. Designing multilevel public health strategies and policy programs may include more frequent contact with lactation specialists in the early postpartum period and effective workplace policies to support breastfeeding for women returning to work.

Our study has several strengths. It included a sufficient sample size that allowed for all statistical analyses with adequate power. The sample was developed randomly and was representative of the target population, which allows generalisability to the current findings. Nevertheless, a few limitations should be acknowledged. The study questionnaire did not include very important questions that could impact the results of the study, such as the type of delivery and physical problems during lactation (mastitis, plugged milk ducts and others). Secondly, we did not analyse an exclusive breastfeeding practice of both groups of mothers, which could be taken into consideration in future studies. Furthermore, there was potential for selection bias in the participants from interviewers who could reject potential respondents from taking part due to appearing to be difficult or living in undesirable locations. The cross-sectional design of the study did not allow us to draw conclusions about causality.

## 5. Conclusions

This study shows several factors positively associated with longer breastfeeding periods (7+ months): socio-demographic factors (higher level of maternal education, nationality, number of children), pregnancy factors (abstaining from smoking during pregnancy) and childbirth factors (initiating breastfeeding immediately after childbirth and on the first day). With this in mind, early and targeted education and empowerment efforts should be directed at mothers at risk of shorter breastfeeding duration, emphasising the importance of early initiation and its association with longer breastfeeding duration.

## Figures and Tables

**Table 1:** Descriptive statistics of characteristics of the study participants (*n* = 1407), weighted data.

Variable	**Length of breastfeeding period**	
	**0–6 months**	**7+ months**
Median	3 months	12 months
	**% (*n*)**	**% (*n*)**
*Socio-demographic characteristics*		
**Nationality of mother (*n* = 1403)**		
Latvian	55.1 (404)	60.7 (409)
Russian	34.1 (250)	26.6 (179)
Other	10.5 (77)	12.5 (84)
**Year of the last labour (*n* = 1390)**		
1970–1999	40.2 (291)	29.9 (199)
2000–2023	59.8 (433)	70.1 (467)
**Income, EURO (*n* = 1277)**		
0–500	63.9 (418)	56.2 (350)
501–800	18.8 (123)	19.6 (122)
≥801	17.3 (113)	24.2 (151)
**Number of childbirths (*n* = 1396)**		
One	36.0 (264)	31.0 (209)
Two	37.1 (272)	46.7 (315)
Three and more	26.9 (197)	22.3 (150)
**Maternal educational level (*n* = 1404)**		
Primary	6.7 (49)	3.1 (21)
Secondary/vocational	55.5 (406)	40.4 (272)
University/college	37.8 (276)	56.2 (380)
*Pregnancy characteristics*		
**Planned pregnancy (*n* = 1342)**		
Yes	78.1 (545)	81.8 (527)
No	21.9 (153)	18.2 (117)
**Smoking during pregnancy (*n* = 1389)**		
Yes	14.4 (104)	5.7 (38)
No	85.6 (619)	94.3 (628)
**Attended prenatal classes (*n* = 1347)**		
Yes	23.5 (164)	32.5 (211)
No	76.5 (534)	67.5 (438)
**Gestational diabetes mellitus and/or hypertension** **(*n* = 1150)**		
Yes	15.2 (90)	14.7 (82)
No	84.8 (504)	85.3 (474)
*Childbirth characteristics*		
**Breastfeed for the first time (*n* = 1383)**		
Immediately after childbirth	53.9 (392)	64.2 (430)
At the first day	28.2 (205)	26.1 (175)
Later	16.1 (117)	9.4 (63)
**Partner’s presence on a labour (*n* = 1401)**		
Yes	21.7 (159)	33.2 (222)
No	78.3 (573)	66.8 (447)
*Postpartum characteristics*		
**Psychological support from partner** **(*n* = 1343)**		
Yes	78.8 (545)	80.8 (526)
No	21.2 (147)	19.2 (125)
**Mother’s need of psychological support** **(*n* = 1237)**		
Yes	37.2 (237)	38.0 (228)
No	62.8 (400)	62.0 (372)
*The sum of responders may differ across variables due to missing values.*		

**Table 2:** Factors associated with longer breastfeeding period in the univariate and multivariate analyses.

	**Duration of** **breastfeeding period** (0–6 months– reference; ≥7 months)		**OR**	**95% CI**	***p*** **-value**	aOR *	**95% CI**	***p*** **-value**
**Variable**	***n***	**%**	**Univariate Analysis**			**Multivariate Analysis**		
*Socio-demographic characteristics*								
**Nationality of mother**								
Latvian	409	50.3	**1.4**	**1.1–1.8**	**0.004**	**1.3**	**1.0–1.7**	**0.03**
Russian	179	41.7	1			1		
Other	84	52.2	**1.5**	**1.0–2.1**	**0.02**	**1.5**	**1.0–2.2**	**0.04**
**Year of the last labour**								
1970–1999	199	40.6	1			1		
2000–2023	467	51.9	**1.6**	**1.3–1.9**	**<0.001**	1.2	0.9–1.7	0.2
**Income (euro)**								
0–500	291	45.8	1					
501–800	181	48.0	1.1	0.8–1.4	0.5			
≥801	151	57.2	1.3	0.9–1.6	0.08			
**Number of childbirths**								
One	209	44.2	1			1		
Two	315	53.7	**1.5**	**1.2–1.9**	**0.002**	**1.5**	**1.1–1.9**	**0.003**
Three and more	150	43.2	1.0	0.7–1.3	0.8	1.0	0.8–1.5	0.7
**Maternal educational level**								
Primary	36	34.3	1			1		
Secondary/vocational	257	40.0	1.6	0.9–2.6	0.1	1.2	0.6–2.3	0.5
University/college	380	57.9	**3.2**	**1.9–5.4**	**<0.001**	**2.0**	**1.0–3.7**	**0.03**
*Pregnancy characteristics*								
**Planned pregnancy**								
Yes	527	49.2	1.3	1.0–1.3	0.08			
No	117	43.3	1					
**Smoking during pregnancy**								
Yes	38	26.8	1			1		
No	628	50.4	**2.8**	**1.9–4.0**	**<0.001**	**2.1**	**1.4–3.3**	**<0.001**
**Attended prenatal classes**								
Yes	211	56.3	**1.6**	**1.2–1.9**	**<0.001**	1.3	0.9–1.7	0.1
No	438	45.1	1			1		
**Gestational diabetes mellitus and/or hypertension**								
Yes	83	47.7	1					
No	501	48.5	1.0	0.7–1.4	0.8			
*Childbirth characteristics*								
**Breastfeeding initiation**								
Immediately after childbirth	430	52.3	**2.0**	**1.5–2.9**	**<0.001**	**1.7**	**1.1–2.4**	**0.01**
At the first day	175	46.1	**1.6**	**1.1–2.3**	**0.01**	**1.6**	**1.2–2.4**	**0.01**
Later	63	35.0	1			1		
**Partner’s presence on labour**								
Yes	222	58.3	**1.8**	**1.4–2.3**	**<0.001**	1.3	0.9–1.7	0.09
No	447	43.8	1			1		
*Postpartum characteristics*								
**Psychological support from partner**								
Yes	526	49.1	1.1	0.8–1.4	0.3			
No	125	46.0	1					
**Mother’s need of psychological support**								
Yes	228	49.0	1					
No	372	48.2	0.9	0.8–1.2	0.8			

Statistically significant results are presented in bold font, *p* < 0.05. Values are weighted. Abbreviations: CI = confidence interval; OR = odds ratio; aOR = adjusted odds ratio. * aOR: adjusted OR, adjustment was performed by including all statistically significant variables mentioned in the table in one model.

## Data Availability

The study data are available on request from the corresponding author. However, due to the presence of sensitive information, they are not publicly available.
